# Cost-effectiveness of comprehensive geriatric assessment at an ambulatory geriatric unit based on the AGe-FIT trial

**DOI:** 10.1186/s12877-017-0703-1

**Published:** 2018-01-31

**Authors:** Martina Lundqvist, Jenny Alwin, Martin Henriksson, Magnus Husberg, Per Carlsson, Anne W. Ekdahl

**Affiliations:** 1 Department of Medical and Health Sciences, Linköping University, Linköping, Sweden; 2 Department of Neurobiology, Care Sciences and Society (NVS), Division of Clinical geriatrics, Karolinska Institute (KI), Stockholm, Sweden; 3 Institution of Clinical Sciences, Lund University, Helsingborg, Sweden

**Keywords:** Cost-effectiveness, Quality-adjusted life years, Comprehensive geriatric assessment, Ambulatory care, Multi-morbidity

## Abstract

**Background:**

Older people with multi-morbidity are increasingly challenging for today’s healthcare, and novel, cost-effective healthcare solutions are needed. The aim of this study was to assess the cost-effectiveness of comprehensive geriatric assessment (CGA) at an ambulatory geriatric unit for people ≥75 years with multi-morbidity.

**Method:**

The primary outcome was the incremental cost-effectiveness ratio (ICER) comparing costs and quality-adjusted life years (QALYs) of a CGA strategy with usual care in a Swedish setting. Outcomes were estimated over a lifelong time horizon using decision-analytic modelling based on data from the randomized AGe-FIT trial. The analysis employed a public health care sector perspective. Costs and QALYs were discounted by 3% per annum and are reported in 2016 euros.

**Results:**

Compared with usual care CGA was associated with a per patient mean incremental cost of approximately 25,000 EUR and a gain of 0.54 QALYs resulting in an ICER of 46,000 EUR. The incremental costs were primarily caused by intervention costs and costs associated with increased survival, whereas the gain in QALYs was primarily a consequence of the fact that patients in the CGA group lived longer.

**Conclusion:**

CGA in an ambulatory setting for older people with multi-morbidity results in a cost per QALY of 46,000 EUR compared with usual care, a figure generally considered reasonable in a Swedish healthcare context. A rather simple reorganisation of care for older people with multi-morbidity may therefore cost effectively contribute to meet the needs of this complex patient population.

**Trial registration:**

The trial was retrospectively registered in clinicaltrial.gov, NCT01446757. September, 2011.

**Electronic supplementary material:**

The online version of this article (10.1186/s12877-017-0703-1) contains supplementary material, which is available to authorized users.

## Background

Older people with multi-morbidity are increasingly challenging for today’s healthcare, and traditional healthcare organization into organ specific care often fails to meet the complex needs of these patients [1]. With a rapid increase in the population of elderly, healthcare solutions that better meet the needs of these patients by improving longevity and quality of life (QoL) at a reasonable cost are required. One such solution is comprehensive geriatric assessment (CGA), a systematic and holistic approach to the care of older people with multi-morbidities. Although the definition and execution of CGA may differ across applications, general components common to most programs include multidisciplinary teams, regular team-meetings and the use of standardized instruments for medical, functional, psychological and social assessments [1, 2].

CGA in hospital settings has been shown to reduce mortality and institutionalization at 12 months follow-up [3]. Furthermore, comprehensive geriatric care in a hospital setting for elderly patients with hip fractures improved mobility and was cost-effective compared to usual orthopedic care [4]. However, few studies have evaluated CGA in the context of outpatient care, and the lack of cost-effectiveness evidence of CGA in general has been recognized [1, 3, 5–7]. In the *Ambulatory Geriatric assessment – a Frailty Intervention Trial* (AGe-FIT), CGA in outpatient care reduced the number of inpatient days and increased the sense of security for the patients at a 2-year follow-up when compared to usual care [8]. At 3 years, CGA patients lived on average 69 days longer with no differences in short-term healthcare costs [9]. To fully assess the overall value of CGA, the long-term (beyond trial follow-up) impact on ultimate health outcomes such as quality-adjusted life years (QALYs) and costs of CGA has to be assessed [10].

### Aim

The aim of this study is to assess the cost-effectiveness of CGA at an ambulatory geriatric unit, compared to usual care for elderly people with multi-morbidity and high healthcare consumption.

## Method

### Analytical approach and cost-effectiveness

The patient population evaluated comprised elderly people with multi-morbidity and high healthcare consumption according to the AGe-FIT trial [8, 9]. The evaluated treatment strategies were CGA at an ambulatory geriatric unit as in the AGe-FIT trial described below and usual care. Costs and QALYs, weighting each year lived with the quality of life (where 1 is full health and 0 is dead), were evaluated over a lifelong time horizon for each treatment strategy using standard methods of decision-analytic modelling [11], synthesizing two years of trial data from the AGe-FIT trial with other relevant data sources. The primary outcome was the incremental cost-effectiveness ratio (ICER) of CGA compared with usual care, relating the incremental costs of CGA to the incremental health outcome (QALYs):

$$ ICER=\frac{Cos{ts}_{CGA}- Cos{ts}_{Usual\kern0.17em care}}{QALYs_{CGA}-{QALYs}_{Usual\kern0.17em Care}} $$

In addition, cost per life year (without quality adjustment) gain was assessed. Costs and health outcomes were discounted by 3% per annum. All costs were calculated in Swedish kronor (SEK), and converted to 2016 euros (€) using the exchange rate of 1 EUR = 9.47 SEK (year 2016 mean exchange rate). The analysis was performed from the perspective of the public healthcare sector including costs for services in both the Municipality and the County Council.

### The AGe-FIT trial

The main data source for the present study, the AGe-FIT trial, has been reported in detail elsewhere [8, 9, 12]. In brief, the AGe-FIT trial was randomized, controlled, assessor-blinded, and carried out in the Municipality of Norrköping, Sweden during 2011 and 2013. Community dwelling patients, of 75 years or older, having three or more concomitant diagnoses and admitted to the hospital for inpatient care three times or more during the past 12 months, were included. The participants gave written informed consent to participate in the study, in case of cognitive decline a proxy gave informed consent according to the protocol [12]. Participants who gave written consent to participate were randomized to either the intervention group receiving care at the ambulatory geriatric unit (AGU) in addition to usual care, or the control group receiving usual care only. The intervention consisted of CGA provided at the AGU and included an interdisciplinary approach in order to make a person-centered plan for future care [12]. Most often it started with a home-visit from a nurse and a social worker to focus on “caring-problems” such as nutrition, skin-problems, elimination and the social environment. A pharmacist reviewed medication before a visit to a physician in the AGU and, depending on needs, a functional investigation by the occupational and physical therapists. In the usual care strategy, standard healthcare services were used as needed.

The AGe-FIT trial was approved by the regional ethical vetting board at Linköping University (No: 2011/41–31 and No: 2015/6–32), and is registered on http://clinicaltrails.gov (NCT01446757). The study adheres to the CONSORT guidelines [13].

### Decision analytic model

In order to estimate the long-term costs and QALYs and assess cost-effectiveness of the CGA intervention, a simple two-state (Alive and Dead) Markov model was employed [14]. In the model, all patients start in the Alive state. During each year (annual Markov cycle) patients face a risk of dying, and thus transition to the absorbing Dead state. The annual risk of dying was conditional on the assigned treatment strategy and based on the 24-month follow-up data from the AGe-FIT trial. The model was run for 30 cycles to ensure that in effect all patients had reached the absorbing Dead state at the termination of analysis. For each Markov cycle that patients reside in the Alive state, they incurred an annual cost and QALY-estimate. There were no costs and QALYs associated with the Dead state. At the termination of analysis, discounted costs and QALYs were summed over all cycles to estimate per patient mean costs and QALYs for CGA and usual care, respectively.

### Data

An overview of data inputs is provided below. Further information is provided in Additional files 1 and 2.

### Costs

Data on healthcare resource use was collected for primary healthcare, ambulatory care (geriatric and other), inpatient care and municipal services (including use of home help services and nursing home utilization) in the Age-FIT trial by linking trial participant–ID to relevant patient registers [8]. For the first two years (Markov cycles) of the analysis these costs corresponded to the per patient mean cost per treatment strategy observed in the AGe-FIT trial (Table 1). From year three and onwards the costs observed in year two of the AGe-FIT trial were applied to the Alive state. The impact on the final results of extrapolating the observed two-year data over a longer time horizon was explored in sensitivity scenarios. See Additional file 2 for details.

**Table 1:** Costs and quality of life data input

	Year 1		Year 2 to 30	
Model parameter	CGA	Usual care	CGA	Usual care
Costs^a^				
Primary healthcare	1672	1957	1256	1670
Ambulatory geriatric care	2366	0	2302	0.3
Other ambulatory care	2382	2372	1708	1876
Inpatient care	4152	5337	3582	4110
Municipal services^b^	8875	7335	10,146	10,698
Quality of life				
QALY estimate Alive state	0.62	0.63	0.61	0.63

*QALY* Quality Adjusted Life Years, *CGA* Comprehensive Geriatric Assessment

^a^Annual per patient mean costs (EUR) in Alive state. ^b^Municipal services includes home-help services and nursing home

### Quality adjusted life years

A QALY-estimate for the Alive state, per treatment strategy, was based on HRQoL data measured with the EQ-5D-3L instrument in the AGe-FIT trial at baseline, 12 months and 24 months [15]. Patient answers on the EQ-5D-3L instrument were converted to QALY-weights using the widely used UK value set [15]. QALY-estimates for year one and two were estimated by calculating the area under the curve for patients alive at 12 and 24 months (Table 1). The QALY-estimate for year two was applied to the Alive state for year three onwards. Furthermore, an annual reduction (for both treatment strategies) in HRQoL was applied to account for the fact that patients get older with an expected health deterioration. Based on published EQ-5D data on the Swedish general population, the QALY-estimates were adjusted by a decrement of 0.0025 per year [16]. The impact on the final results of adjusting the long-term QALY-estimates, was explored in sensitivity scenarios.

### Mortality

For the first two years of the analysis the mortality risks associated with each treatment were estimated from the AGe-FIT trial, and thus corresponded to the observed mortality in the trial. For the first year of analysis this risk was 11.5% and 13.8% for CGA and usual care, respectively. Corresponding figures for year two were 8.2% and 15.3%. The mortality risks for year three and onwards were estimated by using age-specific mortality rates for the general population in Sweden [17]. See Additional file 1 for details.

### Analysis

The analyzed patient population corresponded to the characteristics of patients in the Age-FIT trial with the mean age of the population in the trial (83 years). Uncertainty in the estimated cost-effectiveness results associated with sampling uncertainty in the estimated input parameter values, was evaluated by employing probabilistic sensitivity analysis [18]. In this analysis, the uncertainty in single-model inputs is propagated through the model, using simulation techniques, so that the uncertainty in the cost-effectiveness results indicates the uncertainty in the decision to implement a treatment strategy, rather than the uncertainty surrounding single model inputs [18]. The probability of the CGA being cost-effective at different threshold values for cost-effectiveness was assessed and reported in cost-effectiveness acceptability curves [19]. The importance of parameters not associated with statistical uncertainty for the final results were investigated in sensitivity analyses. See Additional file 2 for details.

All statistical analyses of Age-FIT trial data were performed in SPSS version 22.0 [20]. The decision-analytic model was programmed and analyzed in Microsoft Excel (Microsoft Corporation, Redmond, Washington DC, USA).

## Results

The CGA strategy was associated with an incremental cost of approximately 25,000 EUR compared with usual care (Table 2), mainly due to additional costs for the CGA care. The CGA strategy was associated with a life year gain of 1.05; the gain in QALYs was 0.54, primarily an effect of the mortality reduction, yielding a cost per QALY of approximately 46,000 EUR for CGA compared with usual care. Without the quality of life adjustment the cost per life-year gained for the CGA strategy was approximately 23,000 EUR.

**Table 2:** Costs, outcomes and cost-effectiveness results

	CGA	Usual care	Difference	ICER (EUR)
Base-case analysis				
Costs (EUR)				
Primary healthcare	7881	8477	– 597	
Ambulatory geriatric care	13,742	1	13,741	
Other ambulatory care	10,827	9695	1132	
Inpatient care	21,854	21,381	473	
Municipal services^a^	59,024	49,095	9929	
Total costs	113,327	88,649	24,678	
Outcomes				
QALY	3.33	2.80	0.54	45,987
Life years	5.52	4.46	1.05	23,400

*QALY* Quality Adjusted Life Years, *CGA* Comprehensive Geriatric Assessment, *ICER* Incremental cost-effectiveness ratio

^a^Municipal services includes home-help services and nursing home

**Fig. 1:**
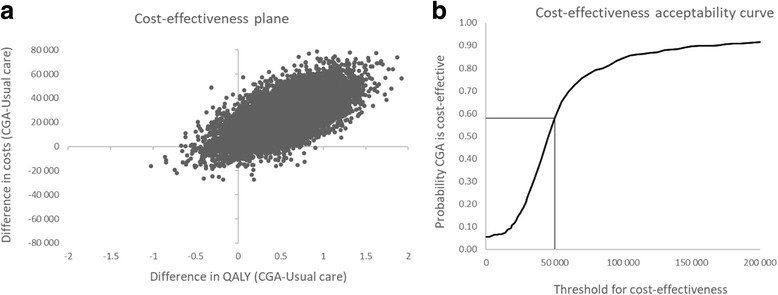
Results of probabilistic analysis. **a** Cost-effectiveness plane based on 10,000 iterations illustrating the distribution of the ICER. **b** Cost-effectiveness acceptability curves showing the probability that CGA is cost-effective at different thresholds for cost-effectiveness

The result of the probabilistic analysis is illustrated on the cost-effectiveness plane in Fig. 1 (panel a). Investigating the joint distribution of incremental costs and QALYs reveals that CGA is associated with an increase in costs in 95% of the simulations (at threshold of cost-effectiveness of 50,000 EUR) and a gain in QALYs in 93% of the simulations. The probability of CGA being cost effective at different threshold values is also shown in Fig. 1 (panel b). At a threshold value of 50,000 EUR this probability was approximately 60% (Fig. 1).

The sensitivity analyses showed small impact on the estimated cost-effectiveness with results ranging from approximately 45,000 to 49,000 EUR. In the scenario when the treatment effect on mortality was set to zero after two years, both the incremental costs and the incremental QALYs decreased, although the impact on overall cost-effectiveness is small. See Additional file 2 for details.

## Discussion

Finding healthcare solutions that meet the needs of older people with multi-morbidity and at the same time are cost-effective is challenging. Assessing the cost-effectiveness of CGA compared with usual care revealed that the CGA is expected to reduce mortality, increase quality-adjusted life expectancy, and increase health care costs. The increased health care costs are primarily a consequence of the intervention costs and costs of increased survival, where patients are assumed to continue to consume health care resources. This contrasts the previous report based on a follow-up of the duration of the Age-FIT trial where it was concluded that the patients lived longer with no difference in short-term health care costs. The full economic evaluation taking a life-long perspective presented here indicates that the health gain is larger than previously reported, and is achieved at an incremental cost, leading to a cost per QALY of 46,000 EUR with CGA compared with usual care.

Previous studies have shown contradicting results when it comes to cost-effectiveness of CGA-based care. When comparing CGA-based care in an orthopedic geriatric unit with usual orthopedic care CGA-based care were found to be both less costly and more effective than usual orthopedic care [4]. Similarly a study concluded that the Dutch Geriatric Intervention program (DGIP) was effective at a reasonable cost for frail older people when compared with usual care [5]. On the contrary, a geriatric medical intervention in an acute setting showed no effect on QALYs and increased costs during a three-month trial follow-up compared with standard care [6]. It should be noted that the setting and study population differs between studies, as does the exact content of the interventions, and the results should therefore be compared with great caution.

A strength of the present study is that it is based on a randomized clinical trial where patients were followed for more than two years with a well-defined and representative study population. Another strength is that registries were used to collect resource use, cost and mortality data reflecting a clinical practice setting. A third strength is that long-term extrapolation of health outcomes and costs beyond the duration of the trial was employed appropriately to estimate cost-effectiveness. Regarding limitations, there was considerable missing HRQoL data in the AGe-FIT study. In a previous analysis of this data, a number of sensitivity analyses tested different replacement methods and showed no major differences in the results [8]. Although the extrapolation of study data to a lifetime time horizon increases the relevance of the results for healthcare decision making, it also introduces inevitable uncertainty. There is always a tradeoff between the relevance and the precision in the estimated cost-effectiveness. In the present study, data from the AGe-fit trial was used in combination with assumptions and data external to the trial for the long-term extrapolation. We believe that the assumptions for the long-term extrapolation were conservative, and sensitivity analyses indicated that the results are unlikely to be altered substantially due to these assumptions.

The results of the sensitivity scenarios indicate that the estimated cost-effectiveness is likely to be around 46,000 EUR per QALY for the CGA strategy. In Sweden there is no explicit threshold value for cost-effectiveness but approximately 50,000 EUR per QALY is often mentioned when considering reimbursement of pharmaceuticals. In the UK for example, an explicit threshold of 20,000–30,000 GBP is employed. Often, aspects other than cost effectiveness are considered when decisions regarding the allocation of healthcare resources are taken. These aspects include severity of the condition, uncertainty of results and implications for the overall healthcare budget. Taken together the estimated cost per QALY of 46,000 EUR may thus be considered value for money in some jurisdictions, whereas in others it may be borderline or even above generally accepted thresholds.

The AGe-FIT trial was performed in a clinical practice setting and the intervention itself may therefore be subjected to substantial improvement with more experience and knowledge. With such improvements it is likely that the cost-effectiveness will also be more favorable. As the interventions themselves are a moving target, it is also important to have flexible and relevant evaluation methods available for continuous updates when new data become available. This study provides such a framework, and the methods are often used when assessing the cost-effectiveness of new pharmaceuticals, but has, hitherto been scarce when assessing the value of new interventions for older patients with multi-morbidity.

## Conclusions

The present study shows that a reorganisation, and more structured management of the care for older people with multi-morbidity, can improve health outcomes at an acceptable cost in order to meet the needs of this complex patient population.

## Additional files

Additional file 1: Mortality. Annual mortality probabilities applied in the model (DOCX 10284 kb)

Additional file 2: Uncertainty and sensitivity analysis. Detailed parameter estimates used in the model and results of sensitivity analysis (DOCX 23 kb)

## Abbreviations

AGe-FIT: Ambulatory Geriatric Assessment – a Frailty Intervention Trail
AGU: Ambulatory Geriatric Unit
CGA: Comprehensive Geriatric Assessment
DGPI: Dutch Geriatric Intervention Program
ICER: Incremental Cost-Effectiveness Ratio
QALY: Quality Adjusted Life Years
QoL: Quality of Life
