# Supplementary material for: Cost-effectiveness of comprehensive geriatric assessment at an ambulatory geriatric unit based on the AGe-FIT trial
Source: BMC Geriatr. 2018 Jan 31;18:32. doi: 10.1186/s12877-017-0703-1 (PMC5793378; doi:10.1186/s12877-017-0703-1)
Supplement: Supplementary file 1 — Mortality. Annual mortality probabilities applied in the model (DOCX 10284 kb) [file 12877_2017_703_MOESM1_ESM.docx]

# Mortality

For the first two years of the analysis the mortality risks associated with each treatment were estimated from the AGe-FIT trial, and thus corresponded to the observed mortality in the trial. For the first year of analysis this risk was 11.5% and 13.8% for CGA and usual care, respectively. Corresponding figures for year two were 8.2% and 15.3%. The mortality risks for year three and onwards were estimated by using age-specific mortality rates for the general population in Sweden (Figure S1). These risks were adjusted upward to account for the added risk associated with our patient population compared with the general population. Data from the AGe-FIT trial was used to calculate the excess risk over standard mortality. Estimated annual mortality probabilities applied in the analysis are illustrated in figure S1 together with annual mortality probabilities of the general population. The assumption of a continued treatment effect beyond 2 years is clearly seen in Figures S1 and S2; the latter illustrating the mortality probabilities with no continued treatment effect beyond 2 years.

**Figure S1. Annual mortality probabilities applied in the model.**

**Figure S2. Annual mortality probabilities applied in the model with no treatment effect beyond 2 years.**
