# Supplementary material for: Cost-effectiveness of comprehensive geriatric assessment at an ambulatory geriatric unit based on the AGe-FIT trial
Source: BMC Geriatr. 2018 Jan 31;18:32. doi: 10.1186/s12877-017-0703-1 (PMC5793378; doi:10.1186/s12877-017-0703-1)
Supplement: Supplementary file 2 — Uncertainty and sensitivity analysis. Detailed parameter estimates used in the model and results of sensitivity analysis (DOCX 23 kb) [file 12877_2017_703_MOESM2_ESM.docx]

# Uncertainty and sensitivity analysis

Detailed parameter estimates used in the model are presented in table S1. QALY-estimates for year one were calculated taking the average of the QALY-estimates measured at baseline and the 12 month follow-up. QALY-estimates for year two were based on the average of the 12-month follow-up and the 24-month follow-up.

**Table S1. Parameter estimates used in the model.**

| Parameter | Mean | Std. Error | Distribution in the probabilistic analysis |
| --- | --- | --- | --- |
| **Comprehensive Geriatric Assessment** | | | |
| *Year 1* |  |  |  |
| Primary Health care | 1 672 € | 199 € | Gamma (71, 224) |
| Ambulatory geriatric care | 2 366 € | 127 € | Gamma (345, 65) |
| Other ambulatory care | 2 382 € | 230 € | Gamma (107, 211) |
| Inpatient care | 4 152 € | 601 € | Gamma (48, 825) |
| Municipal services | 8 875 € | 1 284 € | Gamma (48, 1758) |
|  |  |  |  |
| Mortality | 0.12 | 0.02 | Beta (24, 184) |
|  |  |  |  |
| *Year 2* |  |  |  |
| Primary Health care | 1 256 € | 135 € | Gamma (87, 137) |
| Ambulatory geriatric care | 2 302 € | 160 € | Gamma (206, 106) |
| Other ambulatory care | 1 708 € | 138 € | Gamma (152, 106) |
| Inpatient care | 3 582 € | 648 € | Gamma (31, 1111) |
| Municipal services | 10 146 € | 1 644 € | Gamma (38, 2524) |
|  |  |  |  |
| Mortality | 0.08 | 0.02 | Beta (15, 169) |
|  |  |  |  |
| *QALY-weights* |  |  |  |
| Baseline | 0.62 | 0.02 | Beta (272, 169) |
| 12 month follow-up | 0.62 | 0.03 | Beta (228, 140) |
| 24 month follow-up | 0.60 | 0.03 | Beta (227, 151) |
|  |  |  |  |
| **Usual care** | | | |
| *Year 1* |  |  |  |
| Primary Health care | 1 957 € | 209 € | Gamma (88, 212) |
| Ambulatory geriatric care | - | - | Gamma |
| Other ambulatory care | 2 372 € | 336 € | Gamma (50, 451) |
| Inpatient care | 5 337 € | 698 € | Gamma (58, 865) |
| Municipal services | 7 335 € | 970 € | Gamma (57, 1214) |
|  |  |  |  |
| Mortality | 0.14 | 0.03 | Beta (24, 150) |
|  |  |  |  |
| *Year 2* |  |  |  |
| Primary Health care | 1 670 € | 195 € | Gamma (73, 216) |
| Ambulatory geriatric care | 0.3 € | 0.2 € | Gamma (1, 2) |
| Other ambulatory care | 1876 € | 207 € | Gamma (82, 2179) |
| Inpatient care | 4 110 € | 546 € | Gamma (57, 6879) |
| Municipal services | 10 698 € | 1 419 € | Gamma (57,1781) |
|  |  |  |  |
| Mortality | 0.15 | 0.03 | Beta (23, 127) |
|  |  |  |  |
| *QALY-weights* |  |  |  |
| Baseline | 0.63 | 0.02 | Beta (268, 1609) |
| 12 month follow-up | 0.64 | 0.03 | Beta (208, 116) |
| 24 month follow-up | 0.62 | 0.03 | Beta (171, 103) |

Assumptions required to extrapolate parameters beyond the two years of trial follow-up, including the treatment effect of CGA, and different discount rates, were examined with one-way sensitivity analyses. For example, the assumption of a lifelong treatment effect was investigated by running the model without any differences between the treatment strategies after two years. The results of investigating alternative scenarios are reported in table S2. The sensitivity analyses show small differences in estimated cost-effectiveness, ranging from approximately 45 000 to 49 000 EUR. In scenario five when the treatment effect was set to zero after two years, both the incremental costs and the incremental QALYs decrease, although the impact on cost-effectiveness is small.

**Table S2.** **Results of sensitivity analyses.**

| ***Sensitivity analyses*** | | | | | |
| --- | --- | --- | --- | --- | --- |
| Scenario evaluated |  | CGA | Usual care | Incremental | ICER |
| 1. Discount rate (cost and QALYs): 0% | Cost | 127 718 | 98 073 | 29 646 | 44 811 |
|  | QALY | 3.74 | 3.08 | 0.66 |  |
| 2. Discount rate (cost and QALYs): 5% | Cost | 105 469 | 83 395 | 22 073 | 46 851 |
|  | QALY | 3.11 | 2.64 | 0.47 |  |
| 3. Increase of costs (0.01%) | Cost | 116 434 | 90 621 | 25 813 | 48 102 |
|  | QALY | 3.33 | 2.80 | 0.54 |  |
| 4. No QALY decrement due to age | Cost | 113 327 | 88 649 | 24 678 | 44 790 |
|  | QALY | 3.37 | 2.82 | 0.55 |  |
| 5. Treatment effect set to 0 after 2 years | Cost | 98 418 | 88 649 | 9 769 | 48 577 |
|  | QALY | 3.0 | 2.80 | 0.20 |  |
| QALY=Quality Adjusted Life Years. CGA=Comprehensive Geriatric Assessment. ICER=Incremental cost-effectiveness ratio. †Municipal services includes home-help services and nursing home. | | | | | |
